# An international standardization programme towards the application of gene expression profiling in routine leukaemia diagnostics: the Microarray Innovations in LEukemia study prephase

**DOI:** 10.1111/j.1365-2141.2008.07261.x

**Published:** 2008-09

**Authors:** Alexander Kohlmann, Thomas J Kipps, Laura Z Rassenti, James R Downing, Sheila A Shurtleff, Ken I Mills, Amanda F Gilkes, Wolf-Karsten Hofmann, Giuseppe Basso, Marta Campo Dell’Orto, Robin Foà, Sabina Chiaretti, John De Vos, Sonja Rauhut, Peter R Papenhausen, Jesus M Hernández, Eva Lumbreras, Allen E Yeoh, Evelyn S Koay, Rachel Li, Wei-min Liu, Paul M Williams, Lothar Wieczorek, Torsten Haferlach

**Affiliations:** 1 Roche Molecular Systems, Inc., Department of Genomics and OncologyPleasanton, CA; 2 Moores Cancer Center, University of CaliforniaSan Diego, CA; 3 Department of Pathology, St Jude Children's Research HospitalMemphis, TN, USA; 4 Department of Haematology, University of CardiffCardiff, Wales, UK; 5 Charité, University Hospital Benjamin FranklinBerlin, Germany; 6 Department of Paediatrics, University of PaduaPadua; 7 Division of Haematology, Department of Cellular Biotechnologies and Haematology, University “La Sapienza”Rome, Italy; 8 Institut de Recherche en Biothérapie, CHU de MontpellierMontpellier, France; 9 Munich Leukemia LaboratoryMunich, Germany; 10 Laboratory Corporation of America, RTP, NCUSA; 11 Hospital Universitario de Salamanca and Centro de Investigación del Cáncer (CIC), Universidad de Salamanca-CSICSalamanca, Spain; 12 Departments of Pathology and Paediatrics, National University of SingaporeSingapore

**Keywords:** microarray, gene expression profiling, leukaemia, standardization, diagnostics

## Abstract

Gene expression profiling has the potential to enhance current methods for the diagnosis of haematological malignancies. Here, we present data on 204 analyses from an international standardization programme that was conducted in 11 laboratories as a prephase to the Microarray Innovations in LEukemia (MILE) study. Each laboratory prepared two cell line samples, together with three replicate leukaemia patient lysates in two distinct stages: (i) a 5-d course of protocol training, and (ii) independent proficiency testing. Unsupervised, supervised, and r^2^ correlation analyses demonstrated that microarray analysis can be performed with remarkably high intra-laboratory reproducibility and with comparable quality and reliability.

Several microarray studies have already demonstrated the identification of differentially expressed genes associated with distinct clinical and therapeutically relevant classes of leukaemias (Golub *et al*, 1999; Armstrong *et al*, 2002; Schoch *et al*, 2002; Yeoh *et al*, 2002). Given that microarray assays analyse the expression of multiple genes in parallel, they appear to be a robust test method for diagnostic usage (Kohlmann *et al*, 2003, 2005; Haferlach *et al*, 2005). However, to date, all of these studies aimed at subclassifying leukaemia subtypes through gene expression profiling have been performed mainly as monocentric studies that included only a limited number of patients or using mostly RNA specimens that were predominantly analysed retrospectively from archived samples.

Here we report data from an international study group formed around the European Leukemia Network (ELN, http://www.leukemia-net.org) in 11 laboratories: seven from the ELN, three from the United States, and one in Singapore. The so-called Microarray Innovations in LEukemia (MILE) study programme will prospectively assess the clinical accuracy of gene expression profiles of 16 acute and chronic leukaemia subclasses, of myelodysplastic syndromes (MDS), and a “none of the target classes” control group, as compared to current routine diagnostic workup in over 3000 patients. As a first step representing a major effort to standardize the microarray analysis workflow in the participating centres, a prephase of the MILE study was performed. This report presents the results of the prephase, i.e., a standardization programme of the microarray procedure in the participating laboratories in order to ensure a robust gene expression profiling test performance before patient samples were analysed.

## Materials and methods

There were two stages in the MILE prephase study: protocol training and proficiency testing. As part of the initial protocol training each participating laboratory was provided with identical equipment, including reagent kits, enzymes, spectrophotometer, and heat block instruments, and eight microarray experiments were performed at each centre with an on-site trainer in the respective laboratory being trained. The eight samples analysed during the training course were represented by MCF-7 (breast adenocarcinoma) and HepG2 (liver carcinoma) cell line total RNA (Ambion, Austin, TX, USA) with 1·0 μg and 5·0 μg input of total RNA, respectively, and four leukaemia patient sample lysates prepared from mononuclear cells obtained after Ficoll density purification. Patient lysates comprised cells of one chronic myeloid leukaemia (CML), one chronic lymphocytic leukaemia (CLL), and two replicate lysates of an AML patient sample (containing a translocation t(8;21), French-American-British (FAB) type M2). The total RNA from the patient lysates was extracted at each centre as part of the training programme, making these samples a test of the entire microarray process workflow post sample acquisition (RNeasy kit, Qiagen, Hilden, Germany). Subsequently, after the training phase and for operator proficiency testing, each laboratory independently performed four microarray experiments each for MCF-7 and HepG2 cell lines with inputs of 1·5 μg, 3·0 μg, 5·0 μg, and 8·0 μg total RNA. In total, 204 microarray profiles were included in the analysis (for details see Appendix SI and SII). The three anonymous replicate patient lysates were provided by the Laboratory for Leukaemia Diagnostics in Munich, Germany. All patients gave their informed consent for participation after having been advised of the purpose and investigational nature of the study. The study design adhered to the tenets of the Declaration of Helsinki and was approved by the ethics committees of the participating institutions before its initiation. Details on the microarray analysis workflow, image analysis, quality reports, as well as statistical methods are given in Appendix SI.

## Results

### Intra-laboratory reproducibility of gene expression analyses

**Fig 1:**
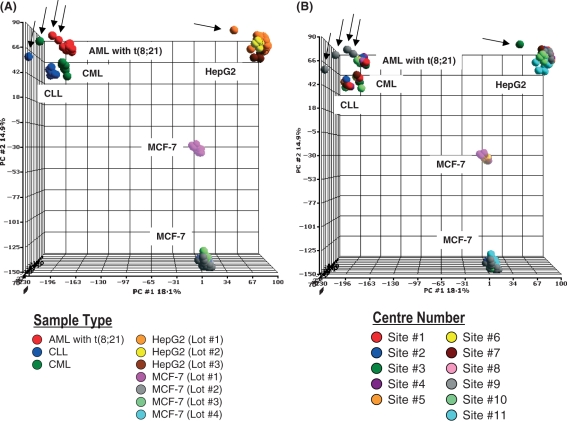
Unsupervised principal component analysis (PCA). A total of 204 experiments are included in the three-dimensional PCA and each sphere represents the gene expression profile for a cell line or leukaemia sample. The signal used is DQN1. The first three principal components (PC) account for 41·0% of variation of the data (PC1 = 18·1%, PC2 = 14·9%, PC3 = 8·0%). The analysis is based on all probe sets represented on the HG-U133 Plus 2.0 microarray without any filtering process (*n* = 54 613). Outliers are marked with arrows. (A) The same sample types are represented by the same colour spheres. Distinct manufacturing batch numbers of the cell lines are given in Appendix SI. (B) Samples processed within the same centre are represented by the same colour spheres.

**Fig 2:**
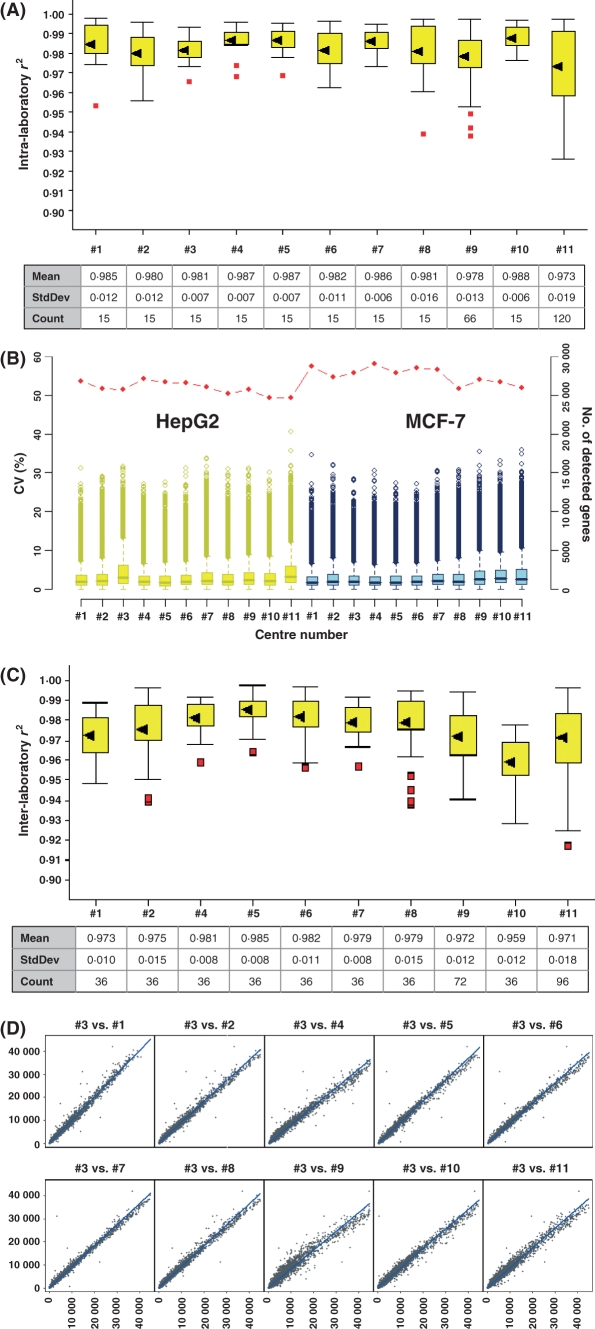
Analysis of intra- and inter-laboratory reproducibility. (A) Box-and-whisker plots display, for each laboratory, the intra-laboratory squared correlation coefficients (r^2^) of all probe sets represented on the HG-U133 Plus 2.0 microarray for the HepG2 cell line sample. The signal used is DS. Each laboratory analysed six HepG2 samples using various amounts of starting total RNA: 1·0 μg, 1·5 μg, 3·0 μg, 5·0 μg (duplicate), or 8·0 μg, respectively. Thus, all possible different pairwise comparisons were performed (Count). Mean r^2^ values (black arrow) and standard deviation (SD) values are given for each of the series of comparisons for each laboratory. Outliers are represented as red boxes. Note: more comparisons were performed in Centres 9 and 11 because multiple operators contributed microarray data (Appendix SII). (B) Repeatability of expression signal within laboratories. The CV of the expression signal values between centre replicates of the same sample type was calculated for all generally detected genes (left *y*-axis). The distributions of replicate CVs are presented in a series of eleven box-and-whisker plots: one for each of the two sample types HepG2 (left) or MCF-7 (right) at the eleven distinct laboratories. The median (line), interquartile range as well as the 10th and 90th percentile values are indicated in each plot. Only genes that were generally detected were included in the box plots and CV calculations. The number of generally detected genes was defined as being called present in at least one third of the samples, e.g., at least two out of the six replicates per centre. This number varied by sample and laboratory and is noted as the line plot with the *y*-axis on the right. (C) Box-and-whisker plots display the inter-laboratory squared correlation coefficients (r^2^) of all probe sets represented on the HG-U133 Plus 2.0 microarray for the HepG2 cell line sample. The signal used is DS. Each centre analysed six HepG2 samples using various amounts of starting total RNA: 1·0 μg, 1·5 μg, 3·0 μg, 5·0 μg (duplicate), or 8·0 μg, respectively. Here, microarray data from Centre 3 is compared with all other laboratories. Each inter-laboratory analysis with different pairwise comparisons is represented by a single box plot (Count). Mean r^2^ values (black arrow) and standard deviation (SD) values are given for each series of comparisons. Outliers are represented as red boxes. Note: more comparisons were performed in Centres 9 and 11 because multiple operators contributed microarray data (Appendix SII). (D) Scatter plot analysis of inter-laboratory reproducibility. The graph shows 10 distinct scatter plot analyses, each displaying a comparison between Centre 3 and the other laboratories for the 5·0 μg HepG2 sample run at the stage of proficiency testing. The r^2^ value calculation is based on DS intensity signals from all probe sets on the HG-U133 Plus 2.0 microarray.

As shown in an unsupervised Principal Component Analysis (PCA), the individual gene expression profiles grouped closely together with their corresponding biological sample types based on the underlying similarity, but not according to the centre where the microarray experiments were performed (Fig 1). The arrows in Fig 1 indicate that the four leukaemia sample preparations from Centre 9 (N17-20), as well as one HepG2 preparation from Centre 3 (N18) were outliers in the PCA. Large differences in gene expression profiles were also observed with respect to the manufacturing batches for MCF-7 total RNA, but overall, a high level of reproducibility between laboratories was seen when a standardized protocol for microarray analysis was followed by trained operators. According to the unsupervised PCA plots, replicated gene expression profiles of the HepG2 cell line were more biologically homogeneous and not as influenced by manufacturing batch numbers, as seen for MCF-7 cell line replicates. Therefore, replicated profiles of the HepG2 cell line were chosen to further investigate the intra- and inter-laboratory correlations. All centres generated highly reproducible gene expression profiles for this cell line, as shown in the box plot analysis of r^2^ values from all pairwise comparisons within each centre for the sample type HepG2 (Fig 2A), where mean r^2^ values range from 0·973 to 0·988. The slightly higher variability at Centre 11 might be explained by a higher number of operators and replicate analyses than in other centres. Figure 2B shows the intra-site repeatability of microarray data based on quantitative signal values and qualitative detection calls. The number of generally detected genes for each sample type at each centre varied from 24 627–27 075 for HepG2 and 25 841–28 953 for MCF-7. The coefficient of variation (CV) of the quantitative signal values between the intra-site replicates was calculated using the generally detected subset of genes for each sample type HepG2 and MCF-7 at each laboratory. The distribution of the replicate CV measures across the set of detected genes is displayed in a series of box plots. The different laboratories demonstrated similar replicate CV median values of 1·962–3·234% for HepG2 and 1·869–2·864% for MCF-7.

### Inter-laboratory reproducibility of gene expression analyses

As an example of inter-laboratory reproducibility of gene expression analyses, correlations between Centre 3 and all other ten laboratories are given (Fig 2C and D). The degree of correlation was only slightly different to the intra-laboratory reproducibility (Fig 2C). The minimum and maximum mean values were 0·959 and 0·985, respectively. This again demonstrated a high inter-laboratory correlation of HepG2 gene expression profiles and confirms the outstanding performance of microarray analysis in the 11 centres. This high inter-laboratory consistency can be also shown in pairwise scatter plot analyses. The 5·0 μg HepG2 replicate analysis between Centre 3 and other laboratories is shown as an example (Fig 2D). A very tight distribution of gene expression data can be observed along the diagonal line for every paired HepG2 sample. Additional analyses of inter-site correlations for HepG2 subsets across all laboratories, along with hierarchical cluster and principal component analyses, are given in Appendix SI. Furthermore, the online section also contains an analysis of the relative contribution of different sources of both technical and biological variability in gene expression measurements.

## Discussion

Taken together, this study demonstrated that standardizing experimental protocols for microarray analysis and performing a thorough operator training resulted in excellent comparability with respect to both data sets generated within a participating laboratory and across 11 different laboratories in three continents. This extends the observations of a recent across-platform comparison study from the Toxicogenomics Research Consortium (Bammler *et al*, 2005). In particular, and also noted by Bammler *et al* (2005), the standardization of RNA labelling protocols using common procedures was recognized as an important contributor to signal intensity correlations across different laboratories. Our study further shows consistent results when compared with the intra-platform precision demonstrated from three different centres in the recent MicroArray Quality Consortia data (Shi *et al*, 2006).

In conclusion, this standardization effort represented the prerequisite foundation of the first phase of the MILE study, wherein 1889 patients have, thus far, been analysed by whole genome expression microarrays (Haferlach *et al*, 2006). The protocol devised for sample preparation takes only one working day from cDNA synthesis to cocktail hybridization and is easily applicable in a daily routine setting. The standardization of gene expression profiling testing in this way has the potential to offer identical objective diagnostic results in any trained laboratory throughout the world. Thus, microarrays are getting substantially closer to a routine application of gene expression profiling for the diagnosis of leukaemias in the clinical practice.

## Authors’ contributions

AK, LW, TH: design of the study and drafting the article; RL, WML, PMW: statistical analysis and interpretation of data; TJK, LZR, JRD, SAS, KIM, AFG, WKH, GB, MCDO, RF, SC, JDV, SR, PRP, JMH, EL, AEY, ESK: data acquisition, interpretation of data, and article revision. All authors approved the final version submitted for publication.

## Supplementary material

The following supplementary material is available for this article online:

**Appendix SI.** Details on microarray analysis, manufacturing lot numbers of cell lines, and additional information on interlaboratory reproducibility.

**Appendix SII.** Information on microarray quality parameters.

**Appendix SIII.** r^2^ correlation data for MCF-7 cell line data.

**Appendix SIV.** r^2^ correlation data for HepG2 cell line data.

**Appendix SV.** r^2^ correlation data for leukaemia samples data.

The material is available as part of the online article from: http://www.blackwell-synergy.com/doi/abs/10.1111/j.1365-2141.2008.07261.x

(This link will take you to the article abstract).
