# Supplementary material for: An international standardization programme towards the application of gene expression profiling in routine leukaemia diagnostics: the Microarray Innovations in LEukemia study prephase
Source: Br J Haematol. 2008 Sep;142(5):802–7. doi: 10.1111/j.1365-2141.2008.07261.x (PMC2654477; doi:10.1111/j.1365-2141.2008.07261.x)
Supplement: Supplementary file 5 [file bjh0142-0802-SD5.doc]

***Supplementary material***

The document contains additional online information as follows:

[Microarray analysis 2](#__RefHeading___Toc195504625)

[Image analysis and data quality report 3](#__RefHeading___Toc195504626)

[Statistical methods 6](#__RefHeading___Toc195504627)

[Supplementary table S1 8](#__RefHeading___Toc195504628)

[r^2^ correlation analysis results 9](#__RefHeading___Toc195504629)

[Unsupervised hierarchical clustering to illustrate inter-laboratory performance 10](#__RefHeading___Toc195504630)

[Supervised Principal Component Analysis 11](#__RefHeading___Toc195504631)

[Supplementary material on distribution of sources of variation. 12](#__RefHeading___Toc195504632)

[Supplementary material on inter-laboratory reproducibility. 13](#__RefHeading___Toc195504633)

[References 19](#__RefHeading___Toc195504634)

# Microarray analysis

Technical variables such as type of microarray, RNA labelling protocol, or data analysis methods can dramatically affect the comparability of gene expression profiling data sets (Bammle*r et* al, 2005; Irizarr*y et* al, 2005; Staa*l et* al, 2006). In our study, not only reagents but also instruments and equipment used for sample preparation such as heat blocks or the spectrophotometer for quantification of nucleic acids were standardized in all participating laboratories.

In addition, for this study, the investigator at each participating centre designated at least one scientist or experienced research technician for training and subsequent sample testing. All laboratory personnel participating in the study were then trained on the standardized microarray sample processing procedure during a five-day training course in the investigator’s laboratory or core facility. Operators were trained both in the routine use of the assay reagents, equipment, and all study-related procedures which did also include the Affymetrix GeneChip system and software. As a prerequisite for the training, basic experience in working with molecular assays, as well as with the regular Affymetrix target preparation protocol was recommended but not mandatory. In fact, it is interesting to point out that centres #1, #9, #10, and #11 had no previous experience with the Affymetrix GeneChip system, yet their results were absolutely comparable to laboratories that had already generated microarray data.

For each sample preparation, total RNA was converted into double-stranded cDNA by reverse transcription using a cDNA Synthesis System kit including an oligo(dT)_24_ – T7 primer (Roche Applied Science, Mannheim, Germany) and Poly-A control transcripts (Affymetrix, Santa Clara, CA, USA). The generated cDNA was purified using the GeneChip Sample Cleanup Module (Affymetrix). Labelled cRNA was generated using the Microarray RNA target synthesis kit (Roche Applied Science) and *in vitro* transcription labelling nucleotide mix (Affymetrix). The generated cRNA was purified using the GeneChip Sample Cleanup Module (Affymetrix) and quantified using the NanoDrop ND-1000 spectrophotometer (NanoDrop Technologies, Wilmington, DE, USA). For each preparation, 11.0 µg cRNA was fragmented with 5X Fragmentation Buffer (Affymetrix) in a final reaction volume of 25 µL. The incubation steps during the cDNA synthesis, *in vitro* transcription reaction, and target fragmentation were performed using the Hybex Microarray Incubation System (SciGene, Sunnyvale, CA, USA) and Eppendorf ThermoStat plus instruments (Eppendorf, Hamburg, Germany). Hybridization, washing, staining, and scanning protocols, respectively, were performed on Affymetrix GeneChip instruments (Hybridization Oven 640, Fluidics Station FS450, Scanner GCS3000) as recommended by the manufacturer.

# Image analysis and data quality report

Microarray image files (.cel data files) were generated using default Affymetrix microarray analysis parameters (GCOS 1.2 software). For each gene expression profile a detailed data report was generated to define the overall quality of each experiment (Appendix SII). The quality parameters that were monitored besides cRNA total yield and cRNA A_260_/A_280_ ratio included: (i) background noise (Q value), (ii) percentage of present called probe sets (Li*u et* al, 2002), (iii) scaling factor, (iv) information about exogenous *Bacillus subtilis* control transcripts from the Affymetrix Poly-A control kit (*lys*, *phe*, *thr*, and *dap*), and (v) ratio of intensities of 3’ probes to 5’ probes for the housekeeping gene *GAPD*.

Quality thresholds required to pass the panel of operator proficiency testing were: (i) cRNA yield for each target preparation was at least 8.0 μg, (ii) percentage of present called probe sets on the HG-U133 Plus 2.0 microarray of at least 25%, and (iii) ratios of 3’ intensity signals of Poly-A controls, spiked-into the total RNA specimens, P/L (*phe*/*lys*), T/L (*thr*/*lys*), and D/L (*dap*/*lys*) greater than or equal to 1.0.

As summarized in the supplementary Appendix SII, all gene expression profiles with the exception of four leukaemia samples experiments (N17-20) from centre #9 passed the quality criteria, i.e. a cRNA yield >8.0 µg in combination with >25% present called probe sets. In these four sample preparations from centre #9, performed during assay training, a cRNA yield of only 2.3 µg – 7.5 µg was achieved and thus these four preparations failed the quality filter. These samples were nonetheless further processed and hybridized to microarrays, yet the overall gene expression profile demonstrated significantly lower present calls.

Also, the impact of input total RNA quantity on the overall assay performance and consistency across the laboratories at the proficiency stage was examined. Although some centre-specific patterns were observed across the various laboratories, the overall trend was similar across the participating centres for both the breast adenocarcinoma MCF-7 and the liver carcinoma HepG2 cell lines sample preparations. All four concentrations of total RNA input, i.e. 1.5 µg, 3.0 µg, 5.0 µg, and 8.0 µg, resulted in very reproducible data sets with respect to yields of cRNA product, percentage of detectable transcripts, global gene expression measurements represented by the scaling factor, and ratio of intensities of 3’ probes to 5’ probes for the housekeeping gene *GAPD* (Supplementary Fig 1). As already shown in the unsupervised PCA in Fig 1, the 3.0 µg total RNA input HepG2 preparation from centre #3 (N18) shows consistent outlier behaviour which can be explained by the significantly lower percentage of present called probe sets (Supplementary Fig 1B).

***Supplementary Fig 1. Microarray data quality analysis.*** *Line graphs are given for various quality parameters and varying amounts of total RNA input in the cell line sample data collected at proficiency stage. The x-axis represents the four separate total RNA input conditions 1.5 µg, 3.0 µg, 5.0 µg, and 8.0 µg each for the HepG2 and the MCF-7 sample type. The participating centres are coded by colours. (A) cRNA yield in µg (y-axis), (B) Percentage of present called probe sets (%P) on the HG-U133 Plus 2.0 microarray (y-axis), (C) Scaling factor (y-axis), and (D) The ratio of intensities of 3’ probes to 5’ probes for the housekeeping gene GAPD (y-axis). Centre-specific outliers are indicated with arrows. For example, although centre #3 passed the proficiency criteria, the %P for the 3.0 µg HepG2 replicate sample (N18, 26.9% present calls) was substantially lower compared to the average of 42.8% present calls of all other replicates and close to the quality cut-off criteria of >25% present called probe sets. Note: The average of the experimental replicates was used for centres #9 and #11 where multiple operators contributed gene expression data.*

# Statistical methods

Data pre-processing included the summarization to generate probe set level signals for each microarray experiment and was performed using algorithms as described in detail elsewhere (Li*u et* al, 2006). Background (BG) was estimated as average of 2% quartile of fluorescent intensities of Perfect Match (PM) and Mismatch (MM) probes and lower bound (LB) of detectable signal intensity was defined as half of the standard deviation. The original probe intensity was recalculated by assigning the maximum of either LB or BG corrected probe intensity. The signal intensity for each probe pair (D) was computed as the difference between PM and MM or as 1 if it was lower than 1. The signal for each probe set was calculated as 40-90% trimmed mean of natural logarithms of all probe-pair intensities.

The probe set signal (DS) was calculated as follows:

$$DS=G*e^{D}$$

where the scaling factor G of an array was solved in the equation $G=500/mean(e^{D},0.02,0.98)$

Secondly, a normalized signal (DQN1) for each probe set was generated with rank-based non-linear transformation of natural logarithm of D signals to beta-1 reference distribution within a microarray (Li*u et* al, 2006).

To assess intra- and inter-laboratory consistency and reproducibility of gene expression microarray analysis, squared Pearson correlation coefficients (r^2^) of DS signal intensity measurements were computed for all possible pair-wise combinations of replicates within cell line types or leukaemia sample classes. Data visualization and exploratory analysis such as box plots, Principal Component Analysis (PCA), and hierarchical clustering were performed with the R software ([http://www.R-project.org](http://www.R-project.org/)), Partek Genomics Suite (http://www.partek.com), and Spotfire DecisionSite (http://www.spotfire.com/products/decisionsite.cfm) (Eise*n et* al, 1998). For data filtering the modified coefficient of variation ($CV'=\frac{StdDev}{Mean+0.1}$) of normalized DQN1 signals as criteria was applied. To examine sources of variation in gene expression measurements, data was fitted with mixed and nested ANOVA models and analysed using R and Partek statistical packages.

All microarray raw data have been deposited in NCBI’s Gene Expression Omnibus database (GEO, http://www.ncbi.nlm.nih.gov/geo/) and are accessible through GEO Series accession number: GSE11135 (Barret*t et* al, 2005).

# Supplementary table S1

**A.** Manufacturing lot numbers of cell lines

|  | **Manufacturing lot** | **Number in manuscript** |
| --- | --- | --- |
| **MCF-7** | 055PO30555B | ***lot #1*** |
|  | 075PO52329B | ***lot #2*** |
|  | 105PO55509A | ***lot #3*** |
|  | 124PO44836A | ***lot #4*** |
| **HepG2** | 035PO41658D | ***lot #1*** |
|  | 065PO36893A | ***lot #2*** |
|  | 125PO54621A | ***lot #3*** |

**B.** Manufacturing lot numbers of cell lines and distribution according to laboratories

**MCF-7 lot number HepG2 lot number**

**Centre #1**: 124PO44836A 035PO41658D

**Centre #2**: 124PO44836A 035PO41658D

**Centre #3**: 124PO44836A 035PO41658D

**Centre #4**: 055PO30555B 035PO41658D

**Centre #5**: 055PO30555B 035PO41658D

**Centre #6**: 055PO30555B 035PO41658D

**Centre #7**: 055PO30555B 035PO41658D

**Centre #8**: 055PO30555B 035PO41658D

**Centre #9**: 075PO52329B 035PO41658D

**Centre #10**: 075PO52329B 065PO36893A

**Centre #11** OT^#^: 124PO44836A 035PO41658D

**Centre #11** PT*: 105PO55509A 125PO54621A

* OT = operator training during training visit

^#^ PT = proficiency testing after training visit

# r^2^ correlation analysis results

Development of a standardized microarray analysis procedure followed by a strict training and proficiency protocol enforced here allowed further data comparisons within and between participating centres. r^2^ correlation analysis is a useful indicator to assess the comparability of microarray experiments either within a centre (intra-site) or between different centres (inter-site). Thus, the r^2^ metrics for MCF-7, HepG2, and sample replicate aliquots from three distinct leukaemia classes were determined. From this analysis it can be seen that the four leukaemia sample preparations from centre #9 (N17-20) are outliers with lower correlation coefficients. Nevertheless, correlation coefficients were ≥0.90 for all microarray experiments that met the pre-set quality criteria limits. In detail, the cell line correlation coefficients ranges, mean value, and standard deviation (StdDev) were 0.905 – 0.998 (mean 0.982, StdDev 0.012) for MCF-7 manufacturing batch 055PO30555B and 0.905 – 0.998 (mean 0.973, StdDev 0.016) for all other MCF-7 manufacturing batches (Appendix SIII), as well as 0.900 – 0.998 (mean 0.972, StdDev 0.016) for HepG2 (Appendix SIV). For the patient samples the correlation coefficients ranges, mean value, and standard deviation were 0.960 – 0.997 (mean 0.985, StdDev 0.009) for CML, 0.964 – 0.998 (Mean 0.984, StdDev 0.007) for CLL, and 0.952 – 0.998 (mean 0.987, StdDev 0.007) for the AML with t(8;21) replicates (Appendix SV). Overall, these results demonstrate a high reproducibility of gene expression measurements generated by 11 different centres, as well as by different sample preparation operators. Moreover, no differences were observed between the training phase and the proficiency testing phase.

# Unsupervised hierarchical clustering to illustrate inter-laboratory performance

The five different sample types are segregated first into distinct clusters at the highest hierarchy level, indicating that between-sample type variability is much greater than inter-laboratory variability (Supplementary Fig 2).

***Supplementary Fig 2. Unsupervised hierarchical clustering analysis.*** *The analysis is based on 6,099 coefficient of variation filtered probe sets of the HG-U133 Plus 2.0 microarray for the 204 experiments included in the study (CV greater than 35%). The signal used is DQN1. Euclidean distance was used to compute the dissimilarity of samples and Ward’s method was used for clustering. The three major clusters that were identified by the algorithm represent HepG2 and MCF-7 with different manufacturing batches, as well as replicates of leukaemia patient samples. The dendrogram with leukaemia patient samples is then further split into AML with t(8;21) (red), CML (green), and CLL (blue). High abundant transcripts with normalized expression level above 0.3 are* *coloured in red and low expressed genes are coloured in green. The overall median expression level is 0.15.*

# Supervised Principal Component Analysis

The segregation of gene expression profiles depending on the sample type rather than the performing laboratory becomes even more obvious when a supervised PCA is performed. As shown in Supplementary Fig 3A, 400 top-ranked differentially expressed probe sets clearly separated sample types by three-dimensional PCA. In the patients’ sample group, CML samples separated from CLL and AML with t(8;21) and these samples also clustered separately from the cancer cell lines. Importantly, Supplementary Fig 3B, that indicates centre distribution, demonstrates that the replicates for each patient sample can be found in close proximity to each other.

***Supplementary Fig 3. Supervised analysis using differentially expressed genes.*** *In the PCA measurements of 200 samples are included. Four samples from centre #9 (N17-20) that failed the quality filtering criteria are excluded. The signal used is DQN1. The analysis is based on 400 top-ranked differentially expressed genes that were identified in a supervised way (ANOVA) to distinguish between the five distinct sample types MCF-7, HepG2, CLL, CML, and AML with t(8;21). A sphere represents each sample’s gene expression profile using the 400-gene signature. The first three principal components (PC) account for 92.8% of variation of the data (PC1=55.7%, PC2=28.1%, PC3=9.0%). (A) Distinction by sample type: spheres with the same colours represent the same sample type. (B) Distinction by laboratory: spheres are coloured according to the location of the laboratory.*

# Supplementary material on distribution of sources of variation.

To assess the relative contribution of variation in gene expression measurements an ANOVA mixed model was applied to fit the normalized microarray data. This model was used to partition any observed variability in the data, according to both technical parameters and different biological sources. As given in the Supplementary Fig 4 the biological sample type accounted for more than half of the variability in the data, followed by the manufacturing batch of the MCF-7 cell line. The differences between laboratories and between different operators in these laboratories contributed only marginally to the overall variability in the gene expression profiles.

***Supplementary Fig 4. Variance components.*** *The chart indicates the various degrees of contributions of different sources to the overall variation in 200 whole-genome gene expression measurements across the 11 different laboratories. Four samples from centre #9 (N17-20) that failed the quality filtering criteria are excluded. The individual contributions of the different sources of variability were estimated with an ANOVA model. The parameters that were analysed included sample type with manufacturing batch number, sample preparation stage (training week vs. proficiency test), operator, input amount of total RNA, and participating laboratory.*

# Supplementary material on inter-laboratory reproducibility.

Box-and-whisker plots are displaying the inter-laboratory squared correlation coefficients (r^2^) of all probe sets represented on the HG-U133 Plus 2.0 microarray for the HepG2 cell line sample. The signal used is DS. Each centre analysed 6 HepG2 samples using various amounts of starting total RNA: 1.0 µg, 1.5 µg, 3.0 µg, 5.0 µg (duplicate), or 8.0 µg, respectively. Microarray data from each centre is compared with all other laboratories. Each inter-laboratory analysis with different pairwise comparisons is represented by a single box plot (Count). For each of the series of comparisons mean r^2^ values (dark blue line) and standard deviation (StdDev) values are given in the table. Outliers are represented as circles. Note: more comparisons were performed in centres #9 and #11 due to the fact that multiple operators contributed microarray data.

# References

Bammler,T., Beyer,R.P., Bhattacharya,S., Boorman,G.A., Boyles,A., Bradford,B.U., Bumgarner,R.E., Bushel,P.R., Chaturvedi,K., Choi,D., Cunningham,M.L., Deng,S., Dressman,H.K., Fannin,R.D., Farin,F.M., Freedman,J.H., Fry,R.C., Harper,A., Humble,M.C., Hurban,P., Kavanagh,T.J., Kaufmann,W.K., Kerr,K.F., Jing,L., Lapidus,J.A., Lasarev,M.R., Li,J., Li,Y.J., Lobenhofer,E.K., Lu,X., Malek,R.L., Milton,S., Nagalla,S.R., O'malley,J.P., Palmer,V.S., Pattee,P., Paules,R.S., Perou,C.M., Phillips,K., Qin,L.X., Qiu,Y., Quigley,S.D., Rodland,M., Rusyn,I., Samson,L.D., Schwartz,D.A., Shi,Y., Shin,J.L., Sieber,S.O., Slifer,S., Speer,M.C., Spencer,P.S., Sproles,D.I., Swenberg,J.A., Suk,W.A., Sullivan,R.C., Tian,R., Tennant,R.W., Todd,S.A., Tucker,C.J., Van Houten,B., Weis,B.K., Xuan,S., & Zarbl,H. (2005) Standardizing global gene expression analysis between laboratories and across platforms. *Nat.Methods*, **2**, 351-356.

Barrett,T., Suzek,T.O., Troup,D.B., Wilhite,S.E., Ngau,W.C., Ledoux,P., Rudnev,D., Lash,A.E., Fujibuchi,W., & Edgar,R. (2005) NCBI GEO: mining millions of expression profiles--database and tools. *Nucleic Acids Res.*, **33**, D562-D566.

Eisen,M.B., Spellman,P.T., Brown,P.O., & Botstein,D. (1998) Cluster analysis and display of genome-wide expression patterns. *Proc.Natl.Acad.Sci.U.S.A*, **95**, 14863-14868.

Irizarry,R.A., Warren,D., Spencer,F., Kim,I.F., Biswal,S., Frank,B.C., Gabrielson,E., Garcia,J.G., Geoghegan,J., Germino,G., Griffin,C., Hilmer,S.C., Hoffman,E., Jedlicka,A.E., Kawasaki,E., Martinez-Murillo,F., Morsberger,L., Lee,H., Petersen,D., Quackenbush,J., Scott,A., Wilson,M., Yang,Y., Ye,S.Q., & Yu,W. (2005) Multiple-laboratory comparison of microarray platforms. *Nat.Methods*, **2**, 345-350.

Liu,W.M., Li,R., Sun,J.Z., Wang,J., Tsai,J., Wen,W., Kohlmann,A., & Williams,P.M. (2006) PQN and DQN: algorithms for expression microarrays. *J.Theor.Biol.*, **243**, 273-278.

Liu,W.M., Mei,R., Di,X., Ryder,T.B., Hubbell,E., Dee,S., Webster,T.A., Harrington,C.A., Ho,M.H., Baid,J., & Smeekens,S.P. (2002) Analysis of high density expression microarrays with signed-rank call algorithms. *Bioinformatics.*, **18**, 1593-1599.

Staal,F.J., Cario,G., Cazzaniga,G., Haferlach,T., Heuser,M., Hofmann,W.K., Mills,K., Schrappe,M., Stanulla,M., Wingen,L.U., van Dongen,J.J., & Schlegelberger,B. (2006) Consensus guidelines for microarray gene expression analyses in leukemia from three European leukemia networks. *Leukemia*, **20**, 1385-1392.
